# Hindered amine light stabilizers in indoor dust: method development and occurrence

**DOI:** 10.1007/s00216-026-06541-y

**Published:** 2026-05-05

**Authors:** Gabriela Castro, Pablo Pereiro, Ysabel Santos, Mauricio Perín, Isaac Rodriguez

**Affiliations:** 1 Department of Analytical Chemistry, Nutrition and Food Sciences. Aquatic One Health Research Center (ARCUS), Universidade de Santiago de Compostela, 15782 Santiago de Compostela, Spain; 2 Department of Microbiology and Parasitology, Aquatic One Health Research Center (ARCUS), Universidade de Santiago de Compostela, 15782 Santiago de Compostela, Spain; 3 Institute of Chemistry, University of Campinas (UNICAMP), Josué de Castro Street S/N, Campinas, 13083-970 Brazil

**Keywords:** HALS, Indoor dust, Human exposure, Tinuvin 770, Tinuvin 292

## Abstract

Hindered amine light stabilizers (HALS) are polymer additives extensively used to improve the durability of plastic materials by inhibiting degradation induced by ultraviolet radiation. Due to their effectiveness, HALS are incorporated into a wide variety of polymeric products intended for both indoor and outdoor applications. However, because these compounds are not chemically bound to the polymer, they can be released into the environment through processes such as volatilization, abrasion, and dissolution. As a result, HALS may accumulate in dust and other environmental matrices. Their occurrence in indoor and outdoor dust raises concerns regarding environmental persistence and potential human exposure, underscoring the need for robust and sensitive analytical methods for their determination. In this study, a new analytical methodology for the determination of HALS in dust samples was developed and optimized. Different sample preparation techniques, including matrix solid-phase dispersion (MSPD), pressurized liquid extraction (PLE), and ultrasound-assisted extraction (UAE), were evaluated with the aim of improving extraction efficiency while minimizing matrix effects. Quantitative analysis was performed using liquid chromatography coupled to tandem mass spectrometry (LC–MS/MS) equipped with a triple quadrupole (QqQ) mass analyzer, providing high sensitivity and selectivity. The optimized procedure was subsequently applied to dust samples collected from various indoor environments to investigate the occurrence and distribution of HALS. These findings contribute to a better understanding of HALS contamination in dust and associated human exposure pathways.

**Graphical abstract:**

**Figure:**
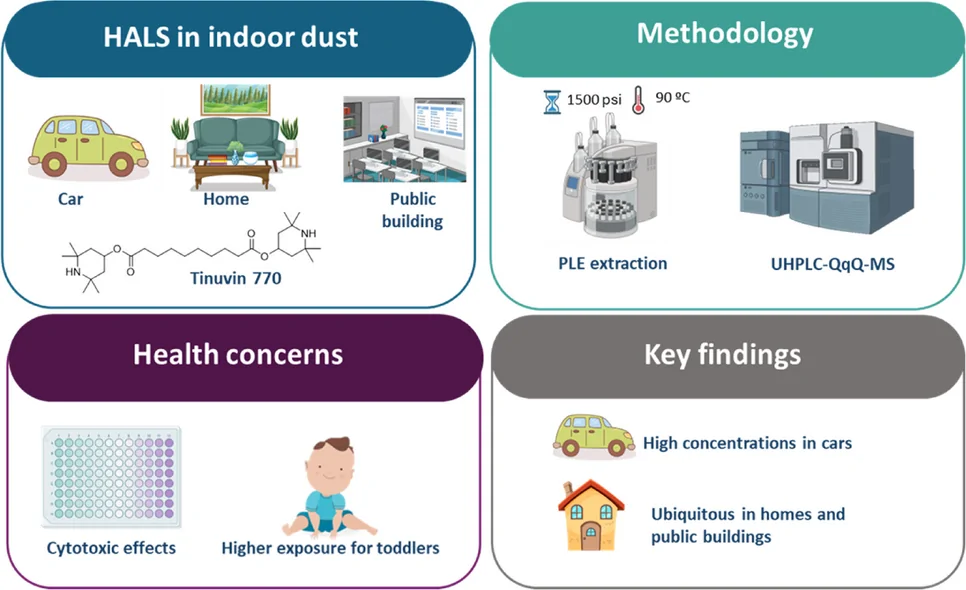

**Supplementary Information:**

The online version contains supplementary material available at https://doi.org/10.1007/s00216-026-06541-y.

## Introduction

Hindered amine light stabilizers (HALS) are organic compounds commonly used as polymer additives to extend material lifespan [1]. Their chemical structure is characterized by a secondary or tertiary amine group surrounded by bulky substituents, which provides steric hindrance against reactive species. This configuration enhances their resistance to oxidation, photodegradation, and thermal degradation, making them highly effective stabilizers [1]. Due to their strong antioxidant properties and capacity to neutralize free radicals through a regenerative mechanism even at low concentrations (≈0.1% w/w), HALS are incorporated in a wide range of applications, including plastics, coatings, textiles, agricultural films, automotive components, and other outdoor-exposed materials, significantly extending their useful life [2–4]. The selection of HALS type and concentration depends on polymer composition, processing conditions, and intended use. In plastic materials, the most widely used stabilizers include Tinuvin 770 and the polymeric Chimassorb 944, both belonging to the second generation of HALS. These compounds are typically employed in a concentration range between 0.1 and 1% w/w and are highly effective in polyolefins (polyethylene, PE; polypropylene, PP) and styrenics (polystyrene, PS; acrylonitrile-butadiene-styrene, ABS). More advanced polymer-bound and N-alkoxy (NOR) HALS, such as Tinuvin 622 and Tinuvin 123, offer superior permanence, low migration, and higher thermal and acid stability, making them suitable for demanding applications in polyamides, polyurethanes, and coatings [3].

However, despite their benefits, the widespread use of HALS raises concerns regarding their presence and persistence in the environment. As these substances are not chemically bound to polymers, they may leach via volatilization, abrasion, or dissolution, ultimately accumulating across different environmental compartments. Recent studies have reported the detection of HALS in indoor dust, air particles, and sediments [2, 5]. Compared to other emerging contaminants, the environmental occurrence and distribution of HALS have not yet been systematically investigated. Indoor dust is a particularly relevant matrix, as it reflects both indoor air quality and human exposure via ingestion, inhalation, and dermal contact [6]. Adults are estimated to ingest between 25 and 50 µg of dust daily, making this pathway especially significant in assessing exposure to contaminants.

As for toxicity, the debate regarding the hazard of several HALS remains open. Within the European Regulatory Framework, several initiatives have been developed to identify and prioritize substances that are prone to reach environmental natural sources, such as the Watch List of substances suspected of posing environmental risks but for which insufficient monitoring data are available [7]. In parallel, growing concern regarding plastic pollution has led to additional policy actions addressing chemical additives associated with plastics [8, 9]. For instance, the REACH Regulation and the work of the European Chemicals Agency (ECHA) have progressively evaluated additives and stabilizers used in polymers, while recent European initiatives targeting microplastics have highlighted the need to better understand chemicals associated with plastic materials and their potential environmental release [10]. Particularly for HALS, they are not included in any list, but ECHA has classified certain representatives, including Tinuvin 114 and Tinuvin 770, as substances of concern due to their potential to cause visual impairment, developmental malformations, and aquatic toxicity [11]. Experimental studies have demonstrated dose-dependent toxic effects of Tinuvin 770 across multiple species and exposure scenarios. For instance, exposure to 25 nmol Tinuvin 770 resulted in cytotoxic effects in rat cardiac myocytes [12]. Consistent with these in vitro findings, chronic cardiotoxicity was observed in rats following intraperitoneal administration of increasing doses of Tinuvin 770 (1, 10, 100, and 1000 mg) [13]. Dose-dependent hemodynamic and cardiac alterations were also reported in dogs receiving bolus injections of Tinuvin 770 (1–100 μg) over a 3-min period [14]. Acute toxicity has been reported in fish at concentrations below 1 mg L^−1^ [2]. As for humans, cases of dermatitis have been associated with prolonged contact with products containing these stabilizers [15]. These findings suggest adverse effects of HALS on both ecosystems and human health.

To evaluate the occurrence and risks of HALS in complex environmental matrices such as indoor dust, robust analytical methodologies are required [1]. Sample preparation represents a critical step, as it must efficiently isolate the target analytes while minimizing matrix interferences. Different extraction techniques have been applied to dust, including matrix solid-phase dispersion (MSPD), ultrasound-assisted extraction (UAE), and pressurized liquid extraction (PLE), each of them offering specific advantages in terms of simplicity, efficiency, and solvent consumption [2, 16, 17]. For determination, liquid chromatography coupled with tandem mass spectrometry (LC–MS/MS) is the preferred approach, providing high sensitivity, selectivity, and quantitative accuracy. Previous works have successfully applied LC–MS/MS to characterize HALS in polymers and environmental samples, although challenges such as multiple protonation states and contamination problems can affect detection limits [18, 19].

Given the scarcity of systematic data and the potential toxicological implications of these compounds, it is crucial to investigate the presence of HALS in indoor dust. With this background, the main objectives of the present study were (i) to develop and optimize an analytical methodology for the extraction and determination of selected HALS in dust samples; (ii) to investigate the occurrence of these substances in dust; (iii) to study the in vitro toxicity of these substances; and (iv) to establish baseline concentrations for determining human exposures through dust ingestion. By generating reliable occurrence data, this work contributes to a better understanding of human exposure to HALS in indoor environments and provides a basis for future risk assessment.

## Materials and methods

### Reagents and materials

**Fig. 1:**
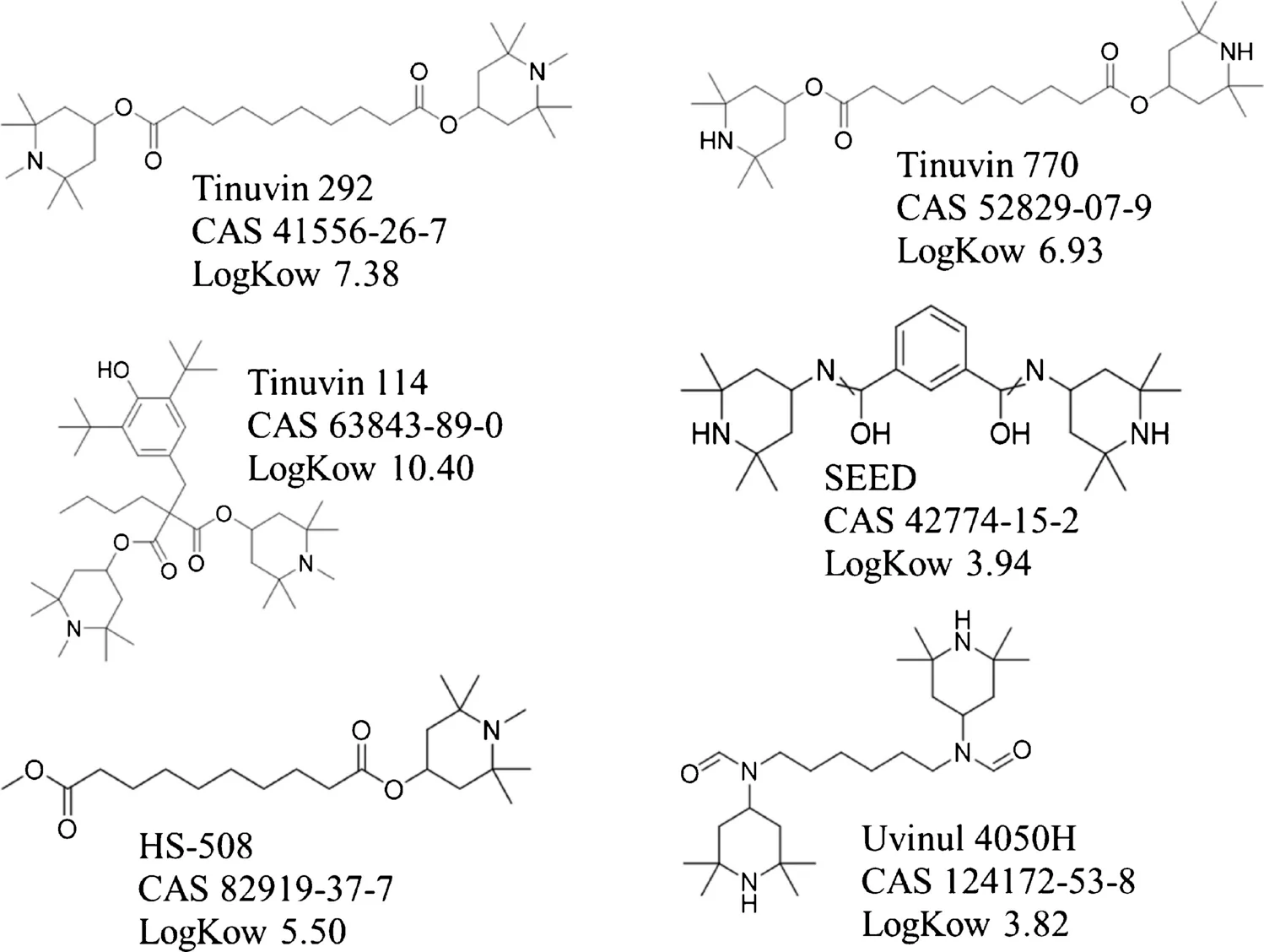
HALS included in this study

Analytical standards of Tinuvin 292 (90%), Tinuvin 114 (98%), Tinuvin 770 (98%), SEED (99.6%), Uvinul 4050 H (97%), and HS-508 (98%) were acquired from Sigma-Aldrich (Milwaukee, WI, USA). Isotopically labelled Tinuvin 770-d_4_ was purchased from TLC Pharmaceutical Standards (Newmarket, Ontario, Canada). Individual solutions of each compound were prepared in methanol (1000 µg mL^−1^). Chemical structures and CAS number of the target HALS are presented in Fig. 1, along with their octanol-water partition coefficients.

Methanol (MeOH), acetonitrile (ACN), water, and formic acid (FA), all of them LC–MS grade quality, were purchased from Fisher Chemicals (Madrid, Spain). Ammonia (NH_3,_ 7 N in MeOH) was obtained from VWR Chemicals (Radnor, PA, USA). Molecular biology grade dimethyl sulfoxide (DMSO) was acquired from Scharlab (Barcelona, Spain), and ultrapure water (18.2 mΩ cm^−1^) was obtained from a Rephile Bioscience Genie equipment (Acton, MA, USA).

As for sorbents, diatomaceous earth was supplied by Sigma-Aldrich, and silica bonded to C_18_ was acquired from Agilent Technologies (Santa Clara, CA, USA).

RPMI 1640 cell culture medium, penicillin-streptomycin, L-glutamine, fetal bovine serum (FBS), and MTT (3-(4,5-dimethylthiazol-2-yl)−2,5-diphenyltetrazolium bromide were purchased from ThermoFisher Scientific S.L. (Madrid, Spain).

### Samples and sample preparation

Dust samples were collected either from domestic vacuum cleaners equipped with cellulose filter bags or manually, from electronic equipment fans, using clean disposable nitrile gloves. In the latter case, dust accumulated in metallic filters, protecting fans of electronic devices, was recovered from the accessible external surface using a brush. To minimize sampling contamination, several measures were implemented: (i) disposable nitrile gloves were replaced between each sample; (ii) samples were individually wrapped and stored in clean aluminum foil; and (iii) the brush was rinsed with water and MeOH between each sampling. After collection, samples were transported to the laboratory, where they were removed from their packaging and sieved to eliminate fibrous materials and organic debris (e.g., hair). The fraction with particle size below 0.22 mm was stored at room temperature in sterile containers until analysis. A total of 28 samples from various indoor environments were analyzed (Table 1).

**Table 1:** Summary of dust samples considered in this study

Sample code	Type	Sampling date
C1	Car	2018
C2	Car	2018
C3	Car	2025
C4	Car	2025
D1	Domestic	2025
D2	Domestic	2025
D3	Domestic	2024
D4	Domestic	2018
D5	Domestic	2018
D6	Domestic	2025
D7	Domestic	2025
D8	Domestic	2018
D9	Domestic	2025
D10	Domestic	2025
D11	Domestic	2025
D12	Domestic	2025
E1	Electronic device filter	2025
E2	Electronic device filter	2025
P1	Public building	2018
P2	Public building	2024
P3	Public building	2024
P4	Public building	2024
P5	Public building	2024
P6	Public building	2025
P7	Public building	2025
P8	Public building	2025
P9	Public building	2025
P10	Public building	2018

Sample preparation conditions for the extraction of HALS from dust were optimized by comparing three extraction techniques: (i) ultrasound-assisted extraction (UAE), (ii) matrix solid-phase dispersion (MSPD), and (iii) pressurized liquid extraction (PLE).

For the UAE, 0.5 g of dust was extracted with 5 mL of MeOH:FA (98:2, v/v) in an ultrasonic bath for 30 min at 35 °C. After centrifugation (10 min, 4000 rpm), the supernatant was collected and transferred into a new PP tube. The extraction was repeated three times, and the supernatants were combined (≈15 mL) followed by concentration to 5 mL under a N_2_ stream. The obtained extract was filtered through a PTFE 0.22-µm hydrophobic filter. As for MSPD, 0.5 g of dust was dispersed in 1 g of C_18_ sorbent using a glass mortar and pestle. The obtained blend was transferred into a PP syringe barrel, containing a PE frit and 1 g of diatomaceous earth. After packing with a second frit, HALS were eluted with 10 mL of MeOH:H_2_O (90:10). The eluate was filtered through a PTFE 0.22-µm hydrophobic filter prior to analysis. Finally, in the case of PLE, 0.5 g of dust was mixed with 1 g of diatomaceous earth and transferred into 11 mL PLE cells containing two additional 1 g layers of diatomaceous earth, below and above the dispersed sample. Extractions were performed with MeOH:FA (98:2, v/v), at 90 ºC and 1500 psi, using two static cycles (5 min each). The flush volume and purge time were set to 100% of the cell volume and 60 s, respectively. The obtained extract (≈25mL) was concentrated to 5 mL under a gentle stream of N_2_ and filtered through a PTFE 0.22 µm hydrophobic filter before UHPLC–MS analysis.

### Instrumental analysis

HALS were quantified using a LC-MS/MS system comprising a Xevo TQD triple quadrupole mass spectrometer equipped with a Z-spray ESI source, coupled to an Acquity UPLC system (Waters, Milford, MA, USA).

Chromatographic separation was achieved on a Zorbax Eclipse Plus C_18_ Rapid Resolution column (2.1 × 50 mm, 1.8 μm; Agilent Technologies, USA) connected to a C_18_ Security Guard™ ultra-cartridge (2.1 mm i.d., Phenomenex, Torrance, CA, USA). Both the analytical column and guard column were maintained at 40 °C throughout the analysis. To prevent Tinuvin 770 contamination derived from the instrument, an InfinityLab PFC delay column (4.6 × 30 mm; Agilent) was installed between the UPLC pump and the injection valve. The mobile phases consisted of (A) LC-MS grade water with 0.1% FA and (B) MeOH with 0.1% FA, delivered at a flow rate of 0.4 mL min^−1^. The chromatographic gradient was programmed as follows: Initial 2% B; 0.5–6.0 min, 100% B; 6.1–7.5 min, 100% B; 7.51–10.0 min, 2% B. The injection volume was 1 µL.

Mass spectrometric detection was performed in positive ESI ionization mode (ESI+) under multiple reaction monitoring (MRM) conditions. Two MRM transitions were monitored for each analyte: the most intense one (higher signal-to-noise, S/N, ratio) was used for quantification (Q1), while the second transition serves as confirmation (Q2) (Table S1). Each transition was monitored within a 90-s window around the retention time of each compound, with a dwell time optimized to achieve 14 points per peak.

High-purity N_2_ (99.999%) was used as drying gas at 450 °C and 800 L h^−1^. The optimized capillary voltage was +1.50 kV, and the cone voltage was set to 50 V.

### Method development

The performance of the different extraction techniques was evaluated through the study of extraction efficiencies (EE%) and matrix effects (ME%) in a pool of dust (composed of six different dust samples). During preliminary evaluation of the different sample preparation protocols, EE% were determined by comparing the peak area of each compound in extracts from spiked dust samples (2000 ng g^−1^) with the peak area obtained from non-spiked extracts that were fortified at the same concentration after sample preparation. The ratio was multiplied by 100 to express the result as a percentage. ME% were calculated as the ratio between the difference in response (peak areas) in spiked and non-spiked extracts of dust and that of a solvent-based standard solution at the same concentration as that added to the extract from dust. Ratios close to 100% indicated negligible matrix effects, while values below or above 100% corresponded to ion suppression or enhancement, respectively [20].

After these preliminary assays, the accuracy and precision of the optimized methodology were evaluated through the study of global recoveries (R%) and ME%. R% were assessed in a pooled dust sample (composed of six different samples) fortified at two concentration levels (2000 and 4000 ng g^−1^) and processed using the optimized protocol. R% were calculated as the ratio between the measured concentration in the extract (after blank subtraction) and the spiked concentration (ng mL^−1^), multiplied by 100. As for ME%, they were evaluated using the ratio between the slopes of matrix-matched calibration curves (extracts spiked at increasing concentrations of HALS) and those of solvent-based standards, multiplied by 100 [20]. Since dust is a complex matrix, ME were dependable on the analyzed samples, so HALS quantification was accomplished based on matrix-matched calibration standards (0 ng mL^-1^, 100 ng mL^-1^, 200 ng mL^-1^, and 300 ng mL^-1^).

The linearity of each compound was evaluated through the analysis of a 10-point solvent-based calibration curve ranging from 1.00 to 1000 ng mL^−1^. Within this range, the obtained graphs followed a linear trend with determination coefficients (*R*^2^) above 0.99 (Table S1). Instrumental repeatability was assessed at three different concentrations (50, 500, and 1000 ng mL^−1^, Table S1), yielding RSD below 14%. Instrumental limits of quantification (iLOQs) were defined as the lowest calibration concentration providing a signal-to-noise ratio of 10. The obtained iLOQs ranged from 1.00 to 10.0 ng mL^−1^ (Table S1). Method quantification limit (mLOQs) of the optimized methodology were calculated as the iLOQs by using pre-extraction spiked pooled samples, also considering the ratio between sample amount (0.5 g) and the final volume of extract (5 mL).

### Quality assurance and quality control (QA/QC)

QA/QC procedures were implemented throughout sample preparation and analysis to prevent contamination and ensure data reliability during the determination of HALS. Specifically, QA/QC measures included (i) thorough cleaning of all laboratory benches and material with acetone prior to use; (ii) two procedural blanks (without dust sample) were prepared per batch of samples to monitor background contamination; (iii) subtraction of blank signals from sample results; and (iv) analysis of solvent blanks and solvent standards (50 ng mL^−1^) every 15 injections to verify carryover and instrumental stability. In addition, all samples were analyzed in duplicate.

### Toxicity tests

The toxicity of HALS was evaluated through in vitro assays, using human HeLa cells (ATCC CCL-2) combined with the MTT (3-(4,5-dimethylthiazol-2-yl)−2,5-diphenyltetrazolium bromide) reduction assay. HeLa cells were cultured at 37 °C, with 5% CO_2_ in RPMI 1640 medium supplemented with 10% fetal calf serum, 100 units mL^−1^ penicillin, 100 mg mL^−1^ streptomycin, and 2 mM L-glutamine. Cells were then seeded on a 96-well cell-culture-treated flat-bottom Microplate (Costar) for 24 h to reach the density of 106 cells mL^−1^ before exposure to increasing concentrations of HALS.

Five concentrations (from 0.005 to 50 µg mL^−1^) of Tinuvin 770 and Tinuvin 292, prepared in DMSO, were added to the wells containing HeLa cells, including five replicates, blank, and control wells on each plate. Cells treated with sterile phosphate-buffered saline (PBS) served as a negative control. Plates were incubated under the conditions described above, and morphological changes were studied using an optical microscope every 3 h for a total HALS exposure time of 24 h. Total or partial destruction of monolayers within a 24 h exposure period was considered a positive cytotoxic effect. After 24 h, the number of viable cells was determined by the MTT viability assays [20]. Briefly, the tissue culture medium was removed from the 96-well plate and replaced with 100 µL of fresh RPMI 1640 medium without phenol red, and then 10 µL of the MTT stock solution (5 mg mL^−1^ in PBS) was added to each well including the control. The 96-well plates were incubated at 37 °C and 5% CO_2_ for 4 h. Optical density was determined by eluting the dye (formazan) with acidic alcohol (hydrochloric acid, 0.04 N solution in isopropyl alcohol), and the spectrophotometric absorbance was measured at 620 nm (Microplate Reader Model 680, BioRad). Experiments were carried out in duplicate. Cells treated with sterile PBS were used as a control for 100% cellular viability. The results were expressed as the percentage of mortality using Eq. 1:

1 $$Normalized\;mortality\;(\%)\:=\:(1\;-\;{Abs}_{Treated}/{Abs}_{Control})\:\times\:100$$

where Abs_Treated_ and Abs_Control_ represent the absorbance values of treated and control samples, respectively.

### Estimation of average daily dose of HALS

Average daily dose (ADD) of HALS through dust ingestion was calculated following the approach described by Christia et al. [21], based on the U.S. EPA exposure assessment framework [22–24] and thus, using Eq. 2:

2 $$ADD\:=\:C_{dust}\;\times\;IngR\;\times\;EF\;\times\;ED\;\times\;ET/(BW\;\times\;AT)$$

where *C*_dust_ is the measured concentration of HALS in indoor dust (ng g⁻^1^); IngR is the daily dust ingestion rate (0.05 g day⁻^1^ for adults and 0.06 g day⁻^1^ for toddlers was assumed as a conservative but realistic estimate [25]); EF is the exposure frequency (350 days year⁻^1^ for cars and 365 days year^−1^ for domestic and public dust); ED is the exposure duration (30 years for adults and 2 years for toddlers); ET is the average daily exposure time in the indoor environment (assumed 1 h day⁻^1^ in cars and 16 h day⁻^1^ in domestic and public indoor environments) for both adults and toddlers; BW is the body weight (70 kg for adults and 12 kg for toddlers); and AT is the average time, calculated as (ED × 350 or 365 days year⁻^1^).

Obtained ADD (ng kg⁻^1^ day⁻^1^) represents the estimated daily intake of HALS via dust ingestion. Parameters related to dermal contact (e.g., skin surface area, adhered dust, and absorption fraction) were not included in this calculation, as only ingestion pathways were considered for HALS exposure assessment.

## Results and discussion

### Background contamination

The principal issue encountered during the analysis of HALS was the background contamination associated with Tinuvin 770. This compound is a widely used plasticizer, present in polymeric components such as laboratory and medical plastics [26], instrument tubing and PEEK fittings, and it is also detected in laboratory dust. To minimize potential contamination, the chromatographic system was thoroughly cleaned with organic solvents, and both the electrospray ionization (ESI) cone and source chamber were washed in accordance with the manufacturer’s recommendations prior to assessing instrumental contamination [27].

**Fig. 2:**
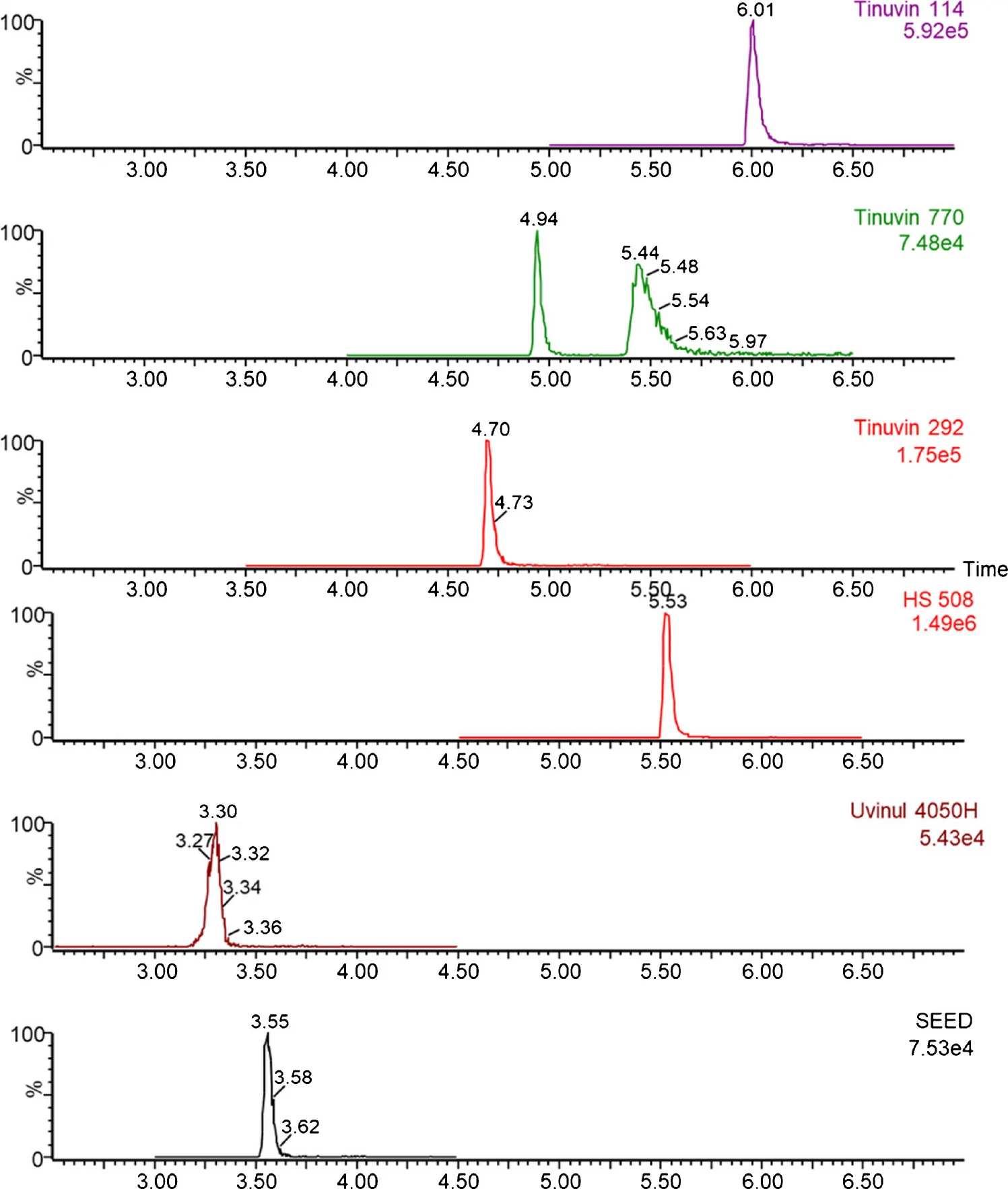
Total ion chromatogram (TIC) of MRM transitions obtained for the studied HALS (standard concentration of 50 ng mL^−1^). The second peak at 5.44 min for Tinuvin 770 corresponds to the contamination coming from the instrument

To differentiate contamination originating from the LC system from the signal in the sample, a delay column was installed between the LC pump and the injection valve. This configuration enabled discrimination between peaks arising from the sample and those attributable to the mobile phase, the latter being observed at slightly longer retention times (Fig. 2). Carryover contamination was assessed by injecting a methanolic standard solution (2000 ng mL⁻^1^) followed by a solvent blank (MeOH). Different washing solutions were evaluated to minimize carryover; among these, a mixture of LC–MS-grade water and MeOH (50:50, v/v) provided the most effective cleaning performance.

To address potential contamination during sample preparation, all laboratory materials were pre-rinsed with organic solvents (MeOH and acetone). In the specific case of PLE cells, components were sonicated for 30 min in a ternary solvent mixture of hexane, acetone, and isopropanol (1:1:1, v/v/v) to remove potential residual contaminants. Procedural blanks (*n* = 2) were included in each analytical batch to monitor background contamination and ensure data quality. Additionally, the use of LC-MS grade water is strongly recommended, as Tinuvin 770 was detected in ultrapure water obtained from the Rephile purification system available in our laboratory.

### Optimization of sample preparation

#### UAE

The sample preparation protocol for the extraction of HALS from dust samples reported by Deng et al. [2] was evaluated with minor modifications (*see *“Samples and sample preparation”). The EE and ME obtained in the UAE are summarized in Table 2. According to the obtained results, the UAE was not suitable for the extraction of HALS from dust, with EE ranging from 16% for Uvinul 4050H to 49% for HS-508. Moreover, substantial signal enhancement was observed, presenting ME ranging from 143% for Tinuvin 114 to 247% for SEED. In all cases, RSD stayed below 21.4%.

**Table 2:** Comparative of extraction efficiencies (EE%) and matrix effects (ME%) with their relative standard deviation (RSD) of the three sample preparation methodologies evaluated for the extraction of HALS from dust. Samples were fortified with 2000 ng g^−1^ (*n* = 3)

Analyte	UAE		MSPD		PLE	
	EE (%, RSD)	ME (%, RSD)	EE (%, RSD)	ME (%, RSD)	EE (%, RSD)	ME (%, RSD)
Tinuvin 292	23 (12)	190 (1.0)	22 (8.0)	132 (22)	93 (6.0)	105 (6.0)
Tinuvin 770	34 (19)	260 (7.4)	26 (8.0)	104 (35)	97 (7.0)	96 (18)
Tinuvin 114	47 (4.6)	143 (21.4)	34 (12)	119 (11)	102 (4.0)	83 (13)
SEED	40 (31)	247 (0.4)	29 (1.0)	84 (15)	92 (14)	66 (49)
HS-508	49 (18)	155 (6.8)	25 (2.0)	95 (8.0)	105 (4.0)	52 (15)
Uvinul 4050H	16 (40)	172 (1.8)	22 (12)	71 (11)	93 (11)	83 (4.0)

To improve extraction selectivity, concentration and compound fractionation experiments were performed using mixed-mode solid-phase extraction (SPE) cartridges combining reverse-phase and cation-exchange functionalities, specifically weak cation exchange (WCX) and strong cation exchange (SCX) sorbents. As for WCX, those compounds presenting a tertiary amino group in their structure (i.e., HS-508, Tinuvin 114, and Tinuvin 292, Fig. 1) presented poor retention in this sorbent. The inefficiency of WCX under acidic conditions was attributed to the protonation of the carboxylic acid functional group, which deactivates the ion-exchange mechanism. In contrast, the SCX cartridge effectively retained all the analytes under acidic conditions; however, quantitative recoveries were not achieved upon elution with MeOH:NH₃ (98:2), and partial degradation of Uvinul 4050H under strongly basic conditions is suspected.

#### MSPD

MSPD methodology was adapted from Carpinteiro et al. [28] with several modifications (*see *“Samples and sample preparation”). To evaluate the performance of this methodology, 2000 ng g^−1^ of the HALS mixture was added to 0.5 g of the pool of dust before the extraction. Obtained EE ranged from 22% for Tinuvin 292 and Uvinul 4050H, to 34% for Tinuvin 114, with RSDs in all cases below 12%. Regarding ME, fortification of target analytes was performed on a final extract of a blank sample. According to the obtained results, MSPD performed better than UAE, showing values between 71% for Uvinul 4050H and 132% for Tinuvin 292 (RSDs below 35%, Table 2). Despite the moderate improvement in ME, the overall extraction performance remained unsatisfactory; therefore, MSPD was excluded from further consideration.

#### PLE

The last sample preparation technique evaluated in this study was PLE. To the best of our knowledge, this is the first time that PLE is employed for the extraction of HALS from dust samples. The optimized extraction procedure is detailed in “Samples and sample preparation.”

Among the evaluated techniques, PLE provided the most satisfactory performance in terms of both EE and ME. In the case of EE, the obtained values ranged from 92 to 105% (RSD < 14), demonstrating quantitative and reproducible recovery of all target analytes (Table 2). ME varied between 52 and 105%, reflecting moderate ionization suppression. Pronounced signal suppression was observed for SEED and HS-508, with ME values of 66% and 52%, respectively. According to the results obtained, PLE demonstrated to perform better than the other studied techniques, displaying quantitative recoveries and lower signal suppression. For all these reasons, PLE was the selected technique for the extraction of HALS from dust.

#### Sample evaporation

Since PLE yields a relatively large extract volume compared to the mass sample (25 mL for 0.5 g of sample), the possibility of concentrating the final extract under N_2_ was considered. Table S2 shows the evaporation losses, corresponding to the 10-fold concentration, 5-fold concentration, and concentration to dryness of an extract of dust. The percentage of losses for a 5-fold concentration ranged from 2.1% to 12% for Tinuvin 114 and Uvinul 4050H, respectively. Concentration to dryness followed by reconstitution of the extract resulted in considerably higher losses, up to 73% for HS-508. Thus, a concentration of 5-fold dust extract was applied, that is, raw PLE extracts were made to a final volume of 5 mL.

### PLE method performance

The ruggedness of the proposed PLE methodology was evaluated through EE and MEs of three different samples, representative of different origins and locations. Thus, samples D4, D5, and C1 were fortified with 2000 ng g^−1^ of the mixture of HALS and submitted to the methodology described in “Samples and sample preparation.”

Obtained EE ranged from 80% to 109%, with RSD below 20%, demonstrating quantitative recoveries for all compounds in the different samples (Table 3).

**Table 3:** EE (%, RSD) obtained for the optimized PLE protocol

Sample	Tinuvin 292	Tinuvin 770	Tinuvin 114	SEED	HS-508	Uvinul 4050H
D4	100 (7)	102 (5)	98 (9)	103 (7)	109 (6)	80 (7)
D5	87 (12)	89 (14)	105 (9)	97 (8)	101 (4)	96 (4)
C1	94 (20)	99 (10)	103 (10)	76 (7)	104 (6)	102 (8)

**Fig. 3:**
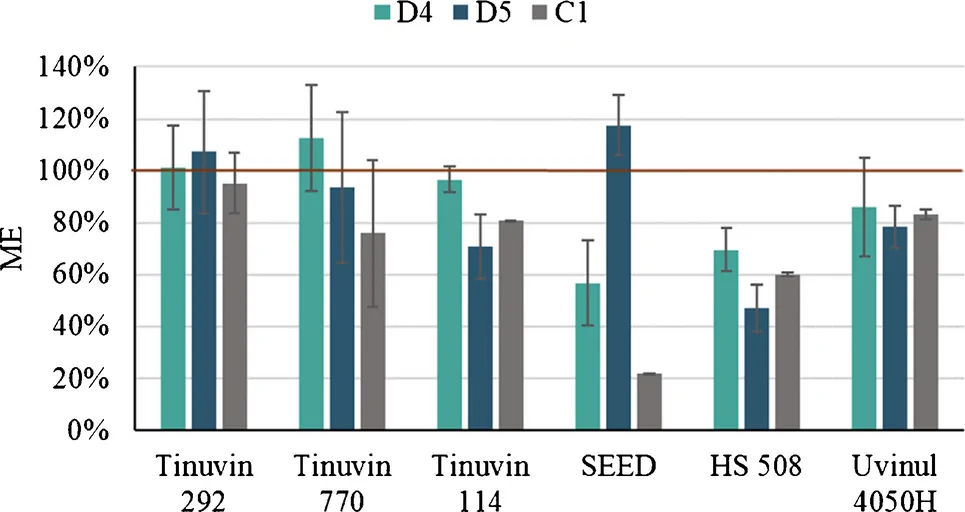
Matrix effects obtained for 3 different dust extracts fortified at 200 ng mL^−1^

In the case of ME, extracts obtained during the extraction of blank samples were fortified with 200 ng mL^−1^ (Fig. 3). Obtained results demonstrated that ME was strongly sample and compound dependent, providing ME between 22% and 118%, with SEED and HS-508 being the substances presenting the highest signal suppression. In case of Tinuvin 770, changes in the efficiency of ionization can be corrected with responses for the deuterated analogue (Tinuvin 770-d_4_); however, the behavior of this compound did not reflect that of the rest of the targeted HALS during ESI ionization (Fig. 3). Also, as far as we could investigate, no deuterated analogues were commercially available for the rest of HALS considered in this research.

Despite these matrix-dependent effects, the overall results confirmed that PLE enables efficient and reliable extraction of HALS from complex dust matrices, making it a promising approach for routine analytical applications. Quantification of dust samples was performed by matrix-matched calibration (fortification concentrations of 100, 200, 300 ng mL^−1^, Fig. S1).

Accuracy of the proposed methodology was assessed by fortifying a pooled sample (composed of six different dust samples) at two different concentrations, 2000 and 4000 ng g^−1^, each processed in triplicate (*see *“Method development”). According to the obtained results, R% varied in the range of 76% to 100% (Table 4).

**Table 4:** Recoveries (%, RSD, *n* = 3 replicates) and method LOQs (ng g^−1^) of the proposed methodology

Analyte	Recoveries (% ± RSD)		mLOQ (ng g^−1^)
	2000 ng g^−1^	4000 ng g^−1^	
Tinuvin 292	93 (13)	80 (11)	50
Tinuvin 770	100 (4.2)	86 (4.2)	50
Tinuvin 114	87 (5.1)	76 (12)	10
SEED	93 (1.8)	91 (2.4)	50
HS-508	83 (5.5)	80 (4.9)	50
Uvinul 4050H	99 (3.0)	90 (5.5)	5

### Analysis of dust samples

The proposed analytical methodology was applied to the collected dust samples, and the obtained concentrations are presented in Table 5. All samples were analyzed in replicates (*n* = 2). The occurrence patterns of HALS in the analyzed samples reveal a widespread distribution of these additives across the different indoor environments. Among the six HALS studied, Tinuvin 770, HS-508, and Tinuvin 292 exhibited the highest detection frequencies (100%, 96%, and 86%, respectively), indicating their wide application in polymeric materials. These HALS are commonly incorporated into plastics, coatings, and textiles to prevent photooxidative degradation, which likely contributes to their persistence and dominance in indoor dust [29]. In the specific case of HS-508 and Tinuvin 292, both compounds are commonly commercialized together as components of the same liquid HALS mixture designed for coating applications [30]. Therefore, the strong relationship observed between their concentrations in dust is consistent with their joint use in commercial formulations and the fact that both substances are emitted simultaneously from treated materials. In contrast, Tinuvin 114, SEED, and Uvinul 4050H were detected in below 30% of the studied samples.

**Table 5:** Concentrations (ng g^−1^) of HALS in the collected dust samples

		Concentration (ng/g)						
		Tinuvin 292	Tinuvin 770	Tinuvin 114	SEED	HS 508	Uvinul 4050H	Total concentration
Car (*n* = 4)	DF (%)	75%	100%	0%	50%	100%	25%	-
	Median	1200	6909	n.d	16187	255	18818	9516
	Min	699	4271	n.d	604	37	18818	4308
	Max	2852	19632	n.d	31771	853	18818	71726
Domestic indoor (*n* = 12)	DF (%)	100%	100%	33%	25%	100%	17%	-
	Median	472	3034	29	99	105	2348	5555
	Min	107	267	10	77	11	112	385
	Max	11156	27366	102	120	1103	4584	28151
Electronic filters (*n* = 2)	DF (%)	50%	100%	0%	0%	50%	0%	-
	Median	359	1321	n.d	n.d	41	n.d	1521
	Min	359	601	n.d	n.d	41	n.d	601
	Max	359	2040	n.d	n.d	41	n.d	2441
Public building (*n* = 10)	DF (%)	80%	100%	20%	30%	100%	20%	-
	Median	777	4820	13	88	62	1762	5547
	Min	224	102	10	65	6	78	394
	Max	36568	13890	16	287	6381	3446	54329
All samples (*n* = 28)	DF (%)	86%	100%	21%	29%	96%	18%	-
	Median	712	4353	16	109	102	3446	5547
	Min	107	102	10	65	6	78	385
	Max	36568	27366	102	31771	6381	18818	71726

*DF*, detection frequency

Across all different environments, car dust presented the highest total median concentrations (9516 ng g^−1^), followed by domestic and public indoor environments (5555 and 5547 ng g^−1^, respectively), and finally dust collected from electronic filters presented the lowest concentrations (1521 ng g^−1^). As for the studied HALS, Tinuvin 770 consistently emerged as the most abundant stabilizer, presenting median concentrations ranging from 1321 ng g^−1^ in dust collected from electronic filters up to 6909 ng g^−1^ in the dust collected from cars (Table S3). Regarding indoor domestic and public environments, the obtained Tinuvin 770 median concentrations were 3034 ng g^−1^ and 4820 ng g^−1^, respectively. The high prevalence of this substance in dust can be attributed to both its stability and its easy potential to migrate from plastic. Tinuvin 292 displayed similar ubiquity but at slightly lower concentrations; median concentrations ranged from 359 ng g^−1^ in electronic filters to 1200 ng g^−1^ in cars, suggesting comparable environmental persistence. The dominance of HALS-type stabilizers over benzotriazole and benzophenone-type UV absorbers (e.g., Tinuvin 114 and Uvinul 4050H) indicates that HALS compounds are more resistant to photolytic degradation and are preferentially retained in indoor dust. The low detection of Tinuvin 114 and Uvinul 4050H (median concentrations of 16 ng g^−1^, DF 21%, and 3446 ng g^−1^, DF 18%, respectively) may also relate to their higher vapor pressures and low volatilization potential, resulting in reduced accumulation in settled dust.

### Variations among environmental compartments

#### Car dust

Car dust samples exhibited the highest concentration, presenting total concentrations ranging from 4308 to 71,726 ng g^−1^. The incredibly high concentration in sample C4 (Table S3) suggests substantial emissions from vehicle interior materials, such as dashboards, seat fabrics, and coatings, which are often exposed to intense sunlight and elevated temperatures. This variability between car sample concentrations may be influenced by different factors such as vehicle use, age, or interior conditions; therefore, no specific sources can be conclusively identified based on the available data.

Tinuvin 770 was consistently the dominant compound, followed by Tinuvin 292 (Table 5), while other analytes were below detection limits in most cases (Table S3). Although indoor pollution in cars has received limited attention, our results corroborate previous findings that automotive environments are significant reservoirs for light stabilizers due to extensive polymer usage and confined air exchange [21, 31]. Regarding the study of HALS in dust from different environments, information in the literature remains scarce. Deng et al. studied the presence of these substances in dust collected from multiple locations. Related to cars, the authors investigated the concentrations of HALS in dust collected from parking lots. The obtained concentrations were lower than those presented here. Tinuvin 770 ranged from 72.9 to 977 ng g^−1^, while Tinuvin 292 stayed between < mLOQ and 142 ng g^−1^. Although these concentrations reflect outdoor rather than in-car dust, they showed the same trend, with Tinuvin 770 presenting the highest concentrations, followed by Tinuvin 292 and HS-508 [2]. Even though this type of sample is not directly comparable to indoor car dust, the reported data provide an indication of the potential magnitude of HALS contamination associated with vehicular sources. As for the concentration of other UV filters detected in indoor car dust, 2-hydroxy-4-methoxybenzophenone (BP-3), 4-methylbenzylidene camphor (4-MBC), homosalate (HMS), and octocrylene (OC) were quantified in car dust samples collected in China [32]. Concentrations reported by the authors for HALS are one order of magnitude lower than those obtained for HALS here. Ao et al. reported median UV filter concentrations between 83 ng g^−1^ for HMS and 326 ng g^−1^ for OC [32].

#### Domestic and public environmental dust

HALS concentrations in domestic and public indoor environments are in the same order of magnitude and present the same trends. Total concentrations ranged from 385 to 28,151 ng g^−1^ for domestic dust, and from 394 to 54,329 ng g^−1^ in the case of public buildings (Table 5). Both environments displayed substantial spatial variability, reflecting differences in household materials, floor coverings, furniture, wall panels, ventilation patterns, and product compositions. Following the same tendency as in car dust, Tinuvin 770 and Tinuvin 292 dominated, presenting median concentrations of 3034 ng g^−1^ and 472 ng g^−1^ in domestic dust and 4820 ng g^−1^ and 777 ng g^−1^ in public buildings (Table 5), reaffirming their environmental persistence and widespread use. Limited detection of Uvinul 4050H and SEED suggests these compounds are less frequently employed in residential products or are more susceptible to degradation and volatilization under indoor conditions. A notable exception was samples D8 and P7 (Table S3), which exhibited an unusually high concentration of Uvinul 4050H (4584 and 3446 ng g^−1^, respectively), likely reflecting localized sources such as treated furnishings or plastic coatings with distinct additive profiles. Deng et al. also studied the presence of HALS in residential houses, reporting lower concentrations than those detected in this study. The authors reported median concentrations of 211 ng g^−1^ of Tinuvin 770 (concentration range 56.9 to 4.04 × 10^3^ ng g^−1^), followed by 64.4 ng g^−1^ of Tinuvin 292 (< mLOQ and 163 ng g^−1^), and 49 ng g^−1^ of HS-508 (4.32 to 216 ng g^−1^) [2] .

#### Electronic device filters

Electronic device filters contained comparatively low total concentrations (601–2441 ng g^−1^), indicating a minor but detectable accumulation of UV stabilizers within electronic components and filtration media. The consistent detection of Tinuvin 292 and Tinuvin 770 aligns with their known applications in electrical housings and polymer-based casings. The presence of these compounds in filter dust also implies their potential release into indoor air, followed by entrapment in ventilation or electronic cooling systems. Deng et al. studied the presence of HALS also in air conditioner filters, reporting similar concentration ranges presented in this study. Aligning with our study, Deng et al. concluded that Tinuvin 770 and Tinuvin 292 were the substances that presented the highest concentrations, between 604 and 1.62 × 10^3^ ng g^−1^ for Tinuvin 770, and between 218 and 437 ng g^−1^ for Tinuvin 292 [2].

### Evaluation of in vitro toxicity

**Fig. 4:**
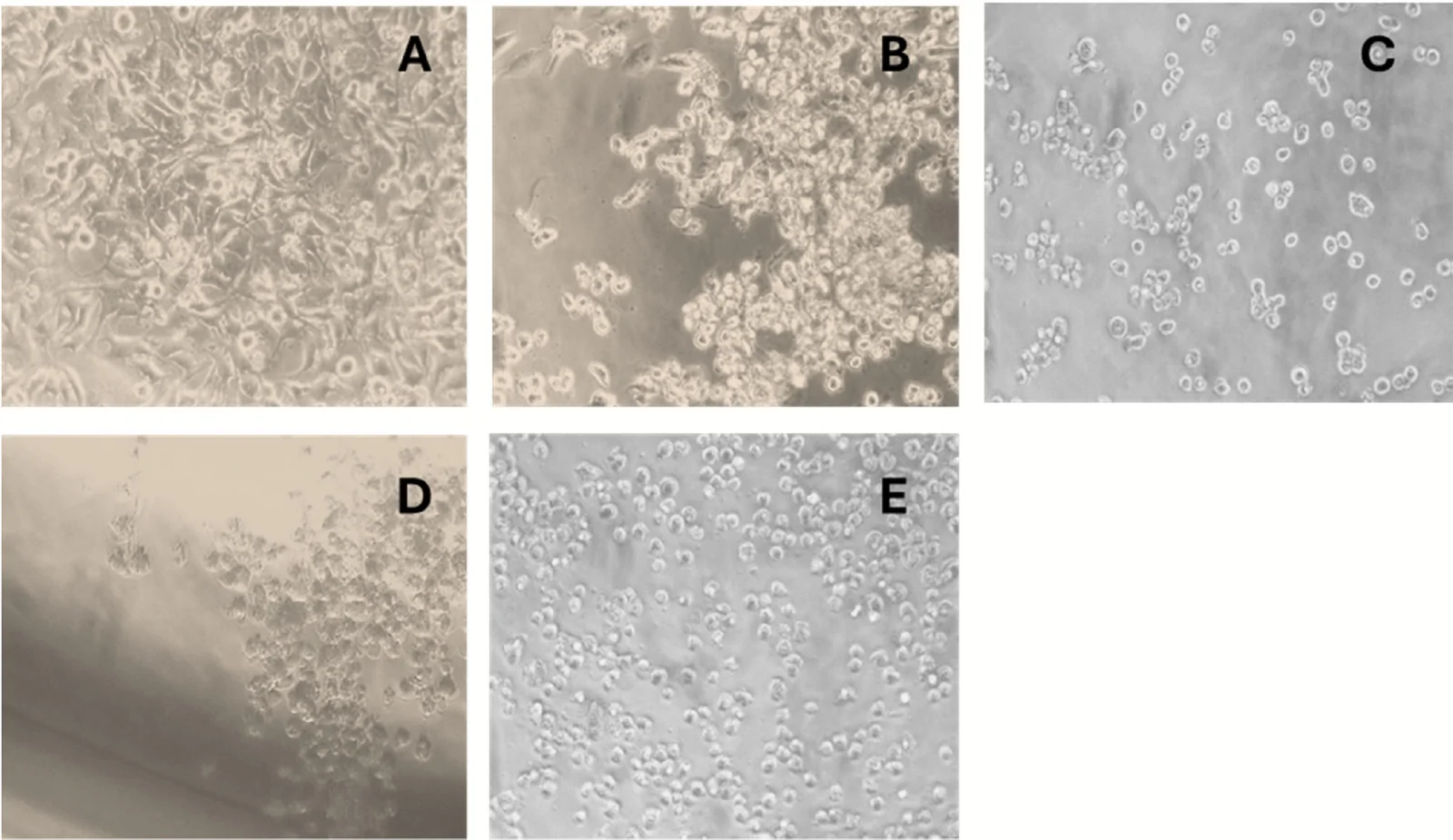
Cytotoxic assay of Tinuvin 770 and Tinuvin 292 on the human HeLA cell line. **A** Control HeLa cells. **B** Initial cytotoxic response manifested by clusters of rounding cells and cell shrinkage after 24 h treatment with Tinuvin 770 (**B**) and Tinuvin 292 (**D**) (50 µg mL^−1^) and final toxic effects manifested by total rounding and cell detachment with Tinuvin 770 (**C**) and Tinuvin 292 (**E**) (5 µg mL^−1^)

The microscopic effect of exposing HeLa cells to increasing concentrations of Tinuvin 770 and Tinuvin 292 is illustrated in Fig. 4. Both substances induced characteristic degenerative morphological changes, including cell rounding, shrinking, and final detachment. These effects were observed after 3–6 h of incubation. The minimal dose necessary to produce monolayer destruction was 5 mg mL^−1^ for both products tested (Figure S2). In contrast, no visible morphological alternations were observed in cells inoculated with lower doses (0.5 to 0.005 mg mL^−1^). Consistent with these observations, MTT assay revealed a reduction in HeLa cell viability of around 40–45% at the highest concentrations studied (50 and 5 µg mL^−1^). Statistical differences in viability between Tinuvin 292 and Tinuvin 770-treated and control HeLa cells were only observed at these doses. Previous studies reported that rat cardiomyocyte cultures exposed to 25 nmol Tinuvin 770 exhibited hypercontraction in 53% of cells after 60 min, while 60% of the cells showed irreversible damage after 120 min of exposure [12]. Moreover, repeated intraperitoneal administration of Tinuvin 770 at doses of 100 to 1000 µg over a 5-week period resulted in histological alterations of the rat myocardium [13].

Taken together, the results of the present study, as well as in vitro and in vivo toxicity data available in the literature [2, 12–14], underscore the need for further investigation into human, animal, and environmental exposure to Tinuvin 770 and the associated health risks. In addition, the relatively high concentrations of Tinuvin 292 in domestic and public indoor environments detected in the present study indicate the need for more information on its potential in vivo toxicity.

### Human exposure via dust ingestion

Human exposure to HALS was evaluated via dust ingestion. For that purpose, average daily doses (ADD) were calculated only for those substances that displayed the highest concentrations and were systematically detected in all samples (Table 5). To that, ADD values were estimated for the different indoor environments (car, domestic, and public buildings) as described in “Estimation of average daily dose of HALS.” Obtained results revealed marked variations among environments and between age groups (Table 6). In all cases, toddlers exhibited substantially higher exposure than adults, which is consistent with previous findings for flame retardants and bisphenols in indoor dust [21, 33].

**Table 6:** Median ADDs (ng kg^−1^ day^−1^) for HALS in different types of dust

		ADD ingestion (ng kg^−1^ b.w. day^−1^)			
		Tinuvin 292	Tinuvin 770	HS-508	∑HALS
Car	Adult	4.60E−02	2.74E−01	1.02E−02	2.62E−01
	Toddler	8.80E−02	5.24E−01	1.94E−02	5.01E−01
Domestic	Adult	6.97E−01	5.80E−02	1.78E−03	7.14E−02
	Toddler	4.91E + 00	2.45E + 01	7.53E−01	3.02E + 01
Public	Adult	1.31E + 00	1.26E + 00	1.26E + 00	1.68E + 00
	Toddler	1.39E + 02	1.33E + 02	2.01E + 01	1.78E + 02

ADD values for ∑_3_HALS ranged from 7.14 × 10^–2^ to 1.68 ng kg^−1^ b.w. day^−1^ for adults, and from 5.01 × 10^–1^ and 178 ng kg^−1^ b.w. day^−1^ for toddlers, which were in the same range as those reported by Deng et al. for adults and toddlers through dust ingestion in China [2]. In the present study, Tinuvin 770 was the main contributor to the ∑_3_HALS intake, which ADD spans from 5.80 × 10^–2^ in cars to 1.33 × 10^2^ public indoor environments.

Despite the relatively low values, these results agree to highlight that the ingestion of settled dust can be a non-negligible exposure pathway for HALS as Deng et al. previously appointed [2], particularly for toddlers. The data also suggest that polymer additives such as Tinuvin 292 and Tinuvin 770 possess higher emission potential than HS-508, possibly due to lower molecular weight and higher volatility.

### Environmental and exposure implications

The widespread detection of HALS in indoor dust across diverse indoor environments underscores their relevance as emerging indoor contaminants associated with polymer-made consumer products. The ubiquitous detection of Tinuvin 770, Tinuvin 292, and HS-508, together with their high concentrations, demonstrates that HALS are readily emitted from treated materials and efficiently accumulate in settled dust, as already documented by Deng et al. [2]. These findings indicate that indoor environments act as important sinks for HALS, driven by continuous emissions from plastics, coatings, textiles, and electronic components.

The incredibly high concentrations detected in car dust (Table 5) underscore the role of vehicles as critical emission hotspots. Elevated temperatures, intense UV radiation, extensive polymer use, and limited air exchange might enhance additive migration and retention inside the vehicle. Given the frequency and duration of human occupancy in vehicles, car interiors may represent a previously underestimated environment for HALS exposure. Similarly, the concentrations measured in domestic and public buildings (Table 5) suggest that everyday indoor settings contribute substantially to background HALS contamination, reflecting widespread use of stabilized polymers in furniture, flooring, wall coverings, and building materials. Their continuous release from consumer products implies that indoor dust may serve not only as a passive reservoir but also as a secondary source, facilitating redistribution through resuspension and subsequent inhalation or ingestion. The detection of HALS in electronic device filters further supports their presence in indoor air and highlights the role of ventilation and cooling systems in capturing airborne additives [2].

From an environmental health perspective, the occurrence of these high concentrations together with the demonstrated in vitro cytotoxicity for Tinuvin 770 and Tinuvin 292 raises concerns about the potential adverse effects associated with chronic exposure to the concentrations detected in dust. Although ADD via dust ingestion were relatively low, toddlers were consistently identified as the most vulnerable group due to higher ingestion rates relative to body weight [21]. This exposure scenario is particularly relevant given the persistence of HALS in dust and the lack of comprehensive toxicological data for several commercially used compounds.

Overall, this study emphasizes the need to consider HALS as environmentally relevant indoor pollutants rather than inert polymer additives. The results support the inclusion of HALS in future indoor exposure assessments, regulatory monitoring programs, and chemical risk evaluations. Further research is warranted to elucidate their long-term fate, transformation products, combined toxicological effects, and contributions from alternative exposure pathways such as inhalation and dermal contact.

## Conclusions

A robust LC–MS/MS analytical methodology was developed and validated for the determination of HALS in indoor dust. The combination of methanol with formic acid, under relatively energetic conditions provided by pressurized liquid extraction, achieved quantitative yields for the extraction of HALS. The analysis of real samples demonstrates the presence of HALS in dust collected from diverse indoor environments.

Tinuvin 770, Tinuvin 292, and HS-508 presented the highest detection frequencies and median concentrations, 4353 ng g^−1^ (detection frequency, DF, 100%), 712 ng g^−1^ (DF 86%), and 102 ng g^−1^ (DF 96%), respectively. Car indoor environments exhibited the greatest contamination (median concentration of 9618 ng g^−1^), identifying vehicles as important indoor emission hotspots, while domestic and public buildings showed comparable concentrations (5555 ng g^−1^ and 5547 ng g^−1^, respectively). Electronic device filters contained lower but consistent HALS concentrations, indicating their presence in indoor air. In vitro assays revealed cytotoxic effects of Tinuvin 770 and Tinuvin 292 over 5 µg g^−1^. Although estimated exposure via dust ingestion was relatively low, toddlers consistently showed higher intake than adults, confirming settled dust as a relevant exposure pathway. Overall, this study highlights HALS as persistent and widespread indoor contaminants and emphasizes the need for further investigation into their toxicological relevance, environmental fate, and inclusion in indoor exposure and risk assessment frameworks.

## Supplementary Information

Below is the link to the electronic supplementary material.

Supplementary file1 (DOCX 55.6 KB)

## Data Availability

The authors declare that the data supporting the findings of this study are available within the paper and its Supplementary Information files. Should any raw data files be needed in another format, they are available from the corresponding author upon request.
