# Supplementary material for: Hindered amine light stabilizers in indoor dust: method development and occurrence
Source: Anal Bioanal Chem. 2026 May 5;418(18):6087–100. doi: 10.1007/s00216-026-06541-y (PMC13541843; doi:10.1007/s00216-026-06541-y)
Supplement: Supplementary file 1 [file 216_2026_6541_MOESM1_ESM.docx]

**Supplementary Material**

**Hindered Amine Light Stabilizers in Indoor Dust: Method Development and Occurrence**

**Gabriela Castro^1^, Pablo Pereiro^1^, Ysabel Santos^2^, Mauricio Perín^1,3^, Isaac Rodriguez^1^**

^1^ Department of Analytical Chemistry, Nutrition and Food Sciences. Aquatic One Health Research Center (ARCUS). Universidade de Santiago de Compostela, 15782, Santiago de Compostela, Spain.

^2^ Department of Microbiology and Parasitology, Aquatic One Health Research Center (ARCUS). Universidade de Santiago de Compostela, 15782, Santiago de Compostela, Spain.

^3^ Institute of Chemistry, University of Campinas (UNICAMP), Josué de Castro Street s/n, Campinas 13083-970, Brazil.

**Journal:** Analytical and Bioanalytical Chemistry (Euroanalysis2025)

***Corresponding author**: Gabriela Castro

Department of Analytical Chemistry, Nutrition and Food Sciences. Aquatic One Health Research Center (ARCUS). Universidade de Santiago de Compostela, 15782, Santiago de Compostela, Spain.

E-mail: [gabriela.c.varela@usc.es](mailto:gabriela.c.varela@usc.es)

**Table S1.** Summary of determination parameters, linearity (R^2^), instrumental repeatability, and limits of quantification (iLOQs) of the UHPLC-MS/MS method.

| ***Compound*** | ***RT*** | ***Cone (V)*** | ***Q1 (CE, V)*** | ***Q2 (CE, V)*** | ***Linearity R^2^ (1.0-1000 ng mL^-1^)*** | ***Repeatability (RSD%, 50ng mL^-1^, n=6)*** | ***Repeatability (RSD%, 500 ng mL^-1^, n=6)*** | ***Repeatability (RSD%, 1000 ng mL^-1^, n=6)*** | ***iLOQ***  ***(ng mL^-1^)*** |
| --- | --- | --- | --- | --- | --- | --- | --- | --- | --- |
| ***Tinuvin 292*** | *4.72* | *40* | *509.4 >72 (35)* | *509.4 > 123 (35)* | *0.9928* | *3.5%* | *5.0%* | *13.8%* | *5.0* |
| ***Tinuvin 770*** | *4.94* | *40* | *481.4 > 140.3 (25)* | *481.4 > 342.3 (25)* | *0.994* | *6.9%* | *4.9%* | *7.6%* | *5.0* |
| ***Tinuvin 114*** | *6.01* | *50* | *685.5 > 72 (45)* | *685.5 > 154.2 (35)* | *0.9939* | *9.0%* | *0.7%* | *4.0%* | *1.0* |
| ***SEED*** | *3.55* | *50* | *443.3 > 123.1 (30)* | *443.3 > 370.4 (25)* | *0.9982* | *3.9%* | *1.3%* | *2.5%* | *5.0* |
| ***HS 508*** | *5.52* | *50* | *370.3 > 72.1 (25)* | *370.3 > 123.4 (25)* | *0.9995* | *4.7%* | *3.2%* | *0.8%* | *5.0* |
| ***Uvinul 4050H*** | *3.2* | *50* | *451.4 > 140.4 (35)* | *451.4 > 123.4 (35)* | *0.998* | *4.9%* | *5.8%* | *1.3%* | *0.5* |
| ***Tinuvin 770-d4*** | *4.95* | *50* | *485.4 > 58.2 (35)* | *485.4 > 140.2 (25)* | *-* | *6.9%* | *8.20%* | *5.50%* | *-* |

**Table S2.** Effect of sample evaporation.

|  | % Evaporation losses | | | | | |
| --- | --- | --- | --- | --- | --- | --- |
|  | **Tinuvin**  **292** | **Tinuvin 770** | **Tinuvin 114** | **SEED** | **HS-508** | **Uvinul 4050H** |
| 10-fold evaporation | 17 (8.1) | 6.1 (4.7) | 1.1 (2.6) | 20 (1.0) | 16 (9.0) | 28 (4.0) |
| 5-fold evaporation | 8.2 (12) | 4.6 (6.3) | 2.1 (3.2) | 11 (5.4) | 3.7 (9.2) | 12 (5.5) |
| Concentration to dryness | 46 (3.4) | 27 (7.3) | 47 (9.7) | 3.2 (0.4) | 73 (24) | 0.1 (1.0) |

**Table S3.** Concentration (ng g^-1^) of HALS in each studied sample.

| **Type** | **Sample code** | **Tinuvin 292** | **Tinuvin 770** | **Tinuvin 114** | **SEED** | **HS 508** | **Uvinul 4050H** | **Total concentration (ng g^-1^)** |
| --- | --- | --- | --- | --- | --- | --- | --- | --- |
| Car dust | C1 | 2852 | 9383 | n.d. | n.d. | 853 | <iLOQ | 13088 |
|  | C2 | <iLOQ | 4271 | <iLOQ | <iLOQ | 37.1 | <iLOQ | 4308 |
|  | C3 | 699 | 4435 | n.d. | 603.7 | 205.1 | n.d. | 5943 |
|  | C4 | 1200 | 19632 | n.d. | 31771.0 | 304.7 | 18817.5 | 71726 |
| Domestic environmental dust | D1 | 2095 | 2796 | <iLOQ | n.d. | 380 | <iLOQ | 5271 |
|  | D2 | 788 | 18891 | n.d. | 98.6 | 108 | n.d. | 19887 |
|  | D3 | 11156 | 4547 | n.d. | n.d. | 1103 | n.d. | 16806 |
|  | D4 | 952 | 2313 | n.d. | <iLOQ | 498 | n.d. | 3762 |
|  | D5 | 138 | 3035 | n.d. | <iLOQ | 22.0 | n.d. | 3195 |
|  | D6 | 221 | 3033 | 16.5 | <iLOQ | 27.0 | <iLOQ | 3298 |
|  | D7 | 759 | 4969 | 41.5 | <iLOQ | 243 | <iLOQ | 6013 |
|  | D8 | 134 | 1021 | 10.0 | 76.7 | 11.4 | 4584 | 5838 |
|  | D9 | 461 | 27366 | 101.7 | 120.1 | 102.2 | n.d. | 28151 |
|  | D10 | 107 | 267 | <iLOQ | <iLOQ | 10.9 | n.d. | 385 |
|  | D11 | 484 | 2297 | <iLOQ | <iLOQ | 150.5 | n.d. | 2931 |
|  | D12 | 388 | 17789 | n.d. | n.d. | 53.9 | 111.9 | 18342 |
| Electronic device filters | E1 | <iLOQ | 601 | <iLOQ | n.d. | <iLOQ | n.d. | 601 |
|  | E2 | 359 | 2040 | n.d. | n.d. | 40.9 | n.d. | 2441 |
| Public buildings | P1 | <iLOQ | 102 | n.d. | 287 | 5.73 | <iLOQ | 394 |
|  | P2 | 36568 | 11380 | n.d. | n.d. | 6381 | n.d. | 54329 |
|  | P3 | 995 | 13890 | n.d. | n.d. | 371 | n.d. | 15256 |
|  | P4 | 411 | 5833 | 15.6 | 64.5 | 56.6 | n.d. | 6381 |
|  | P5 | 363 | 4787 | 10.4 | 87.6 | 45.9 | 78.4 | 5372 |
|  | P6 | 830 | 2537 | n.d. | <iLOQ | 67.2 | n.d. | 3434 |
|  | P7 | <iLOQ | 265 | n.d. | n.d. | 17.1 | 3446 | 3728 |
|  | P8 | 224 | 1081 | <iLOQ | <iLOQ | 53.4 | n.d. | 1358 |
|  | P9 | 1502 | 5319 | n.d. | n.d. | 407.4 | n.d. | 7228 |
|  | P10 | 725 | 4853 | n.d. | n.d. | 145.0 | n.d. | 5722 |
|  | DF (%) | 86% | 100% | 21% | 29% | 96% | 18% |  |

**Figure S1**. Comparative of the slopes obtained for samples D4, D5, C1 and the calibration curve prepared in methanol.

Tinuvin 292

Area

| Concentration (ng mL-1) | C1 | D4 | D5 | Solvent based standard |
| --- | --- | --- | --- | --- |
| 0 | 10489.3 | 5605.22 | 637.572 | 0 |
| 100 | 13917.9 | 12974.1 | 8993.66 | 7016.87 |
| 200 | 19224.4 | 22011.2 | 13469.7 | 12259.5 |
| 300 | 24043.8 | 27129.1 | 18444.6 | 20409.2 |

**Figure S2.** Normalized mortalities of HeLa cells versus Tinuvin 292 and Tinuvin 770 doses. Bars represent the mean ± Standard error. Asterisks indicate significant differences (p < 0.05) in viability between treated and untreated Hela cells.
